# Can child-focused sanitation and nutrition programming improve health practices and outcomes? Evidence from a randomised controlled trial in Kitui County, Kenya

**DOI:** 10.1136/bmjgh-2018-000973

**Published:** 2019-01-05

**Authors:** Gerishom Gimaiyo, Jeffery McManus, Matt Yarri, Shiva Singh, Andrew Trevett, Grainne Moloney, Ann Robins, Lilian Lehmann

**Affiliations:** 1 East Africa Department, IDinsight, Nairobi, Kenya; 2 Technical Team Department, IDinsight, San Francisco, USA; 3 Data Analytics Department, Lyft, San Francisco, USA; 4 WASH Department, UNICEF, Kampala, Uganda; 5 WASH Department, UNICEF, Nairobi, Kenya; 6 Regional Office for Eastern and Southern Africa (ESARO) - Nutrition Department, UNICEF, Nairobi, Kenya; 7 Nutrition Department, UNICEF, Nairobi, Kenya; 8 Southeast Asia Department, IDinsight, Manila, Philippines

**Keywords:** community-led total sanitation (clts), nutrition, stunting, sanitation and hygiene, child health, diarrhoea

## Abstract

**Introduction:**

In Kenya’s Kitui County, 46% of children under 5 years are stunted. Sanitation and nutrition programmes have sought to reduce child undernutrition, though they are typically implemented separately. We evaluate the effectiveness of an integrated sanitation and nutrition (SanNut) intervention in improving caregiver sanitation and nutrition knowledge and behaviours.

**Methods:**

We conducted a cluster-randomised controlled trial to evaluate the impact of the SanNut intervention on caregiver knowledge, sanitary and hygiene practices, sanitation outcomes and nutrition outcomes. The evaluation included caregivers of children under 5 years across 604 villages in Kitui County. 309 treatment villages were randomly assigned to receive both the SanNut intervention and the standard Community-Led Total Sanitation (CLTS) intervention, while 295 control villages only received the CLTS intervention. 8 households with children under 5 years were randomly selected from each evaluation village to participate in the endline survey, for a total of 4322 households.

**Results:**

SanNut led to modest improvements in sanitary knowledge and practices emphasised by the programme. Caregivers in treatment villages were 3.3 pp (+32%) more likely to mention lack of handwashing after handling child faeces as a potential cause of diarrhoea, and 4.9 pp (+7.8%) more likely to report safe disposal of child faeces than caregivers in control villages. Treatment households were 1.9 pp (+79%) more likely to have a stocked handwashing station and 2.9 pp (−16%) less likely to report incidences of child diarrhoea. However, SanNut appears to have had no impact on nutritional practices, such as breastfeeding, vitamin A supplementation or deworming. Non-child outcomes traditionally associated with CLTS, including latrine use and homestead sanitary conditions, were similar in treatment and control groups.

**Conclusion:**

Child-focused messaging can potentially be integrated into CLTS programming, though this integration was more successful for topics closer to CLTS objectives (sanitation practices, including limiting faecal contamination and handwashing) than for more disparate topics (nutritional practices).

**Trial registration:**

Pan-African Clinical Trials Registry (PACTR201803003159346) and American Economic Association registry for randomised controlled trials (AEARCTR-0002019).

**Key messages**

**What is already known?**

- Community-Led Total Sanitation (CLTS) programmes improve access to latrines and reduce instances of open defecation among children and adults. CLTS programmes also increase the availability and use of functional handwashing stations.
- One systematic review of community-based nutrition education programmes finds that these interventions improve the nutrition status of under-five children in low-income and middle-income countries.

**What are the new findings?**

- Delivering a child-focused programme alongside CLTS improves sanitation and hygiene knowledge and practices, especially as they relate to the care of children below 5 years of age.
- Integrating nutrition messages into CLTS did not affect the feeding practices of children below 2 years of age. Since the sanitation and nutrition (SanNut) programme incorporated a limited set of nutrition messages, further research is needed to understand whether other nutrition interventions that address a wider set of nutrition outcomes would have significant impact on child health outcomes.

**What do the new findings imply?**

- The SanNut programme presents an opportunity within CLTS for delivering child-focused sanitation messages, closely linked to overall health outcomes targeted by CLTS.
- As most CLTS-specific behaviour change outcomes remained unaffected by the SanNut intervention, SanNut neither enhanced nor crowded out CLTS messaging on sustained behaviour change of sanitation practices.

## INTRODUCTION

Undernutrition contributes to 45% of all child deaths. Undernutrition can cause child deaths directly (eg, through vitamin A and zinc deficiencies) or indirectly contribute to case fatality (by increasing the risk of mortality from other conditions/diseases).^1^ Approximately, 40% of stunted children live in Africa, which is the only region where the number of stunted children is projected to increase over the next 10 years.^1^

One of the underlying causes of undernutrition in children from low-income and middle-income countries is poor sanitation. Evidence has linked faecal contamination of the environment with environmental enteropathy, a gut disorder that causes malabsorption of nutrients and is associated with stunting in children.^2–4^ In recent years, several community-based interventions aimed at improving rural sanitary practices have grown in popularity. Community-Led Total Sanitation (CLTS), an approach that mobilises communities to eliminate open defecation through latrine construction and behaviour change, has proven to be one of the most common of these types of interventions. A systematic review of household-based studies of community interventions like CLTS (including in combination with other interventions) presents evidence of modest increases in latrine coverage and use,^5^ though another systematic review found varied success in reducing the presence of faeces within the homestead and around the latrine (for instance, a CLTS intervention in Mali and a program promoting safe disposal of child faeces in Nigeria led to lower observation of faeces, whereas there was no reduction in faecal contamination due to India’s Total Sanitation Campaign or a CLTS+sanitation marketing program in Tanzania).^6^ As a result, there is limited evidence that CLTS interventions reduce diarrhoea and weak evidence that CLTS and other sanitation interventions reduce stunting.^7–9^ Given the mixed success of these interventions in improving the community’s approach to sanitation, an open question is whether implementers can leverage CLTS to highlight the link between faecal contamination and child undernutrition, and thereby further improve child-specific sanitary practices and health outcomes.

A separate class of interventions have sought to reduce child undernutrition by promoting healthy nutritional practices. Breastfeeding interventions that employ similar implementation strategies as CLTS—community-wide mobilisation to shift behaviours and norms—have been successful at promoting exclusive and complementary breastfeeding rates in low-income and middle-income country settings.^10–13^ These programmes are often paired with a suite of micronutrients supplementation interventions, including vitamin A, folic acid, iodine and zinc supplementation, to further reduce micronutrient deficiencies. Prior research suggests that deaths of children 5 years and younger can be reduced by 15% by promoting these evidence-based nutrition interventions.^14^

Our study reports the results of one attempt to combine both of these types of behavioural interventions—promoting child-focused sanitary practices and promoting healthy nutrition behaviours—into one programme and deliver it alongside an existing CLTS intervention. CLTS programmes present a natural opportunity for this type of supplementary intervention given their strong community-outreach component and linkages with the local public health system. However, it is unclear if additional child-centred messaging would complement existing activities or ‘crowd out’ CLTS messaging, perhaps by overburdening local health professionals or diluting the message to beneficiaries.

The interventions took place in Kitui County, Kenya, which has one of the highest rates of child malnutrition in the country, and where 46% of children exhibit stunting.^15^ From June 2016 to January 2017, the Kitui County Government implemented a county-wide CLTS programme across 2100 villages as part of a larger preventive and promotive health initiative called the Pamoja Tujikinge Magonjwa Integrated Programme (PATUMAIP). A combined sanitation and nutrition (SanNut) intervention was designed and delivered using CLTS implementation structures from October 2016 to January 2017. The SanNut programme engaged caregivers of children under 5 years through two community meetings and additional messaging during routine household visits about the importance of a sanitary household environment, proper hygiene practices and various nutritional practices (including health-seeking behaviour) to promote child health.

We conducted a cluster-randomised controlled trial (RCT) to measure the impact of this SanNut programme on sanitation and nutrition knowledge and practices. Three hundred and nine treatment villages were randomly selected to receive the SanNut supplemental programme with the CLTS intervention (CLTS+SanNut group), while 295 control villages only received the CLTS intervention (CLTS only group). We find the SanNut programme led to modest improvements in sanitary knowledge and practices, especially safe handling of child faeces and handwashing. However, SanNut appears to have had no short-term impact on nutritional practices. Outcomes traditionally associated with CLTS, including latrine construction and maintenance, were similar in treatment and control villages.

This paper is organised as follows. The Methods section describes the SanNut programme, the RCT design and sample and how data were collected. The Results section reports the causal impact of SanNut on sanitation and nutrition outcomes. The paper concludes with a Discussion section of the results and opportunities for further research.

## Methods

### CLTS and SanNut interventions

Like other CLTS programmes, the CLTS programme implemented in 2016 by the Kitui County Government, focused on helping communities achieve Open Defecation Free (ODF) status. Within each eligible (non-ODF) village, the programme kicked off with CLTS implementers visiting the village to set a date and location for subsequent activities and to map where open defecation occurs. Following this visit, facilitators led a 2–4 hour community-wide meeting (‘triggering’) to stimulate feelings of shame and disgust around sanitation conditions in the village. This meeting included typical CLTS exercises, such as walking through the village to observe where open defecation occurs and encouraging community members to construct and use latrines. Community health volunteers (CHVs) later visited households to reinforce the messages from the triggering event. The CHVs who implemented both CLTS and SanNut were recruited under the PATUMAIP program, which required CHVs to have primary-level education and be at least 30 years old. One CHV was recruited from each village and was responsible for implementing the community health strategy in their village. CHVs worked exclusively in their assigned village and were supervised by the Public Health Officers in their Ward. They were provided with a monthly stipend of Kshs. 3000 (~US$30) to facilitate their work but did not receive any incentive-based payments. There was no gender requirement for CHV recruitment;the CHV workforce consists of roughly similar numbers of men and women. All CHVs attended ward-level CLTS trainings in July 2016, and CHVs in Treatment villages attended an additional SanNut training in September 2016. Villages that successfully achieved ODF status held a community celebration.

The SanNut programme was designed by the Kitui County Government and UNICEF to address recognised gaps in CLTS messaging. SanNut specifically extended the focus of CLTS to children, highlighting the consequences of poor sanitation and nutrition practices on children’s health outcomes and ultimately to the child’s long-term well-being. SanNut’s programming included the topics listed in table 1 that distinguished it from broader CLTS objectives:

**Table 1:** Community-Led Total Sanitation (CLTS) versus sanitation and nutrition (SanNut) topics

CLTS topics	Additional SanNut topics
Highlight all points of open defecation and other sources of faeces within the community that can lead to contamination of everyone in the community. Link diarrhoeal diseases and the associated health costs with poor sanitation. Emphasise the use of latrines and handwashing with water and soap/ash among adults to prevent faecal contamination.	Highlight all sources of faecal matter within the homestead that can lead to faecal contamination of children as they interact with their environment. Link stunting and impaired cognitive development in children to poor sanitary and nutritional practices. Emphasise proper disposal of child faeces and handwashing with water and soap/ash among both children and adults. Promote correct infant feeding practices especially exclusive and complementary breastfeeding at appropriate ages, and encourage the use of nutrient-rich foods. Encourage caregivers to bring children to regular health facility visits in order to receive routine health services, such as vitamin A supplementation and deworming.

Kitui County officials oversaw SanNut programme administration while UNICEF provided technical and financial support. Within randomly selected treatment villages, CHVs invited all caregivers of children under 5 years and pregnant women to participate in SanNut by attending meetings about toddler hygiene and nutrition. These invitations were extended through community leaders, announced in public forums, and in many cases, delivered door-to-door by CHVs. The 1-hour meetings were held at a caregiver’s home or at a public location within the village.

The first SanNut caregiver meeting was held 2–3 weeks after CLTS triggering and focused primarily on toddler hygiene and sanitation. The key message from this meeting was ‘Keep faeces away from infants and infants away from faeces.’ The meeting included a discussion linking faecal ingestion to child malnutrition, disease and impaired cognitive development, discussions of how to safely dispose of child faeces and how to wash the child’s hand before feeding, and an interactive exercise where caregivers developed an action plan for keeping children in safe, hygienic environments. Facilitators referred to a session guide with key SanNut messages and used visuals throughout the meeting, including an ‘F-Diagram’ that depicts faecal–oral pathways, brain scan images that contrast a normal health child’s brain with a malnourished child’s brain and Maternal, Infant and Young Child Nutrition (MIYCN) counselling cards that reinforce proper sanitation and nutrition practices.

The second SanNut caregiver meeting was held 1–3 weeks after the first caregiver meeting and focused primarily on healthy nutritional practices. Facilitators discussed the importance of breastmilk, how caregivers should exclusively breastfeed children below 6 months and how caregivers should complement breastfeeding with solid food consumption for children 6 months to 2 years. Facilitators also explained how children should be taken to health facilities for deworming treatment, vitamin A supplementation and when they are sick. Facilitators referred to a session guide and supporting materials such as MIYCN counselling cards that reinforced proper sanitation and nutrition practices throughout both meetings.

**Figure 1:**
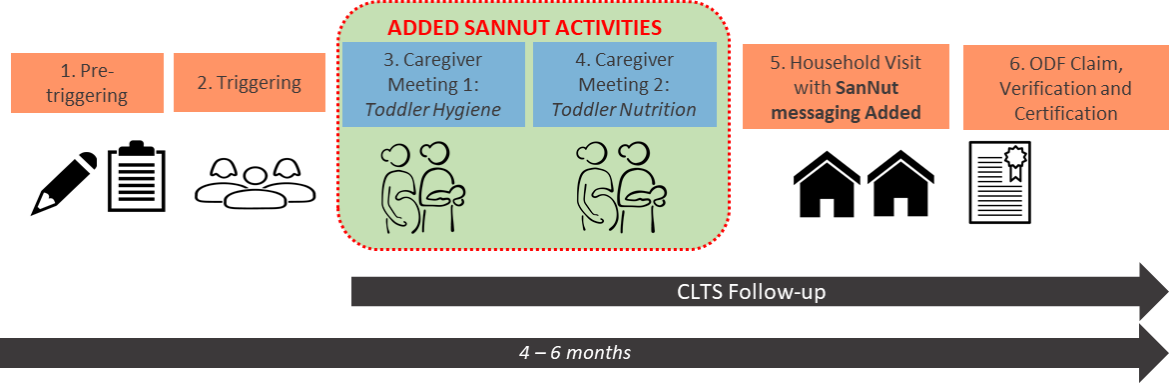
SanNut activities within CLTS implementation in treatment villages. CLTS, Community-Led Total Sanitation; ODF, open defecation free; SanNut, sanitation and nutrition.

In the months following the caregiver meetings, CHVs visited households in the community up to four times to reinforce the messages from the triggering event and caregiver meetings. These household visits occurred in both treatment and control villages, but the messaging in the visits differed depending on the treatment status. In control villages, CHVs discussed the topics covered in the CLTS triggering event and checked whether households had constructed a latrine and were using it and whether the household had a stocked handwashing station. In treatment villages, CHVs covered the same material as in control villages but added SanNut-specific messaging. This additional messaging included the topics from the caregiver meetings, especially the safe disposal of child faeces, keeping infants away from faecal material, appropriate breastfeeding practices and the importance of regular health facility visits. Figure 1 shows how SanNut activities fit into CLTS implementation in a typical village. The SanNut program was relatively low-cost since it took advantage of the infrastructure and personnel in the CLTS intervention. SanNut required US$34 per village for SanNut facilitators to travel to villages and deliver the two caregiver meetings. However, we do not have access to information on the cost of ward-level trainings for facilitators or any other costs associated with their involvement in SanNut. The CHV workforce was hired and funded under the PATUMAIP CLTS program, and since they performed SanNut activities as part of their routine household visits, they did not require additional funding.

### RCT design

We designed a cluster RCT to measure the effects of the SanNut programme on caregiver practices and sanitation and nutrition outcomes. To construct the sampling frame, we applied several eligibility criteria to the 2100 villages slated to participate in the CLTS programme in Kitui County. First, we excluded three of eight subcounties that are mostly urban or periurban and thus retained the five rural subcounties that were more appropriate for the rural-focused CLTS programme. Second, we excluded one ward (the administrative unit below subcounty and above village) that was more than 90% ODF, since it had few villages participating in the CLTS programme. Third, we excluded six wards where Population Services Kenya, an NGO, was implementing a nutrition intervention with many similarities to the SanNut programme. Fourth, we excluded villages that were far from a health facility (more than 10 km) due to the logistical barriers posed to caregivers in taking children to health facilities; we reasoned that we could only measure the effect of the intervention on a household’s demand for health services if those services were accessible. These criteria left 724 villages in our sampling frame.

We conducted power calculations to determine the minimum sample size necessary to detect treatment effects of 0.15 SD or larger, which is near the lower bound of effect sizes of successful sanitation and nutrition programs in our literature review. We used a conservative estimate of 0.2 for the correlation of sanitation and nutrition outcomes between households in the same village, which was the upper bound of intracluster correlations observed in similar studies.^16 17^ Assuming a sample of five households per village, based on village populations and age distributions from census data, we identified a minimum sample size of 520 villages. To account for potential treatment non-compliance or other factors that could reduce statistical power, we inflated our estimates by ~20%, resulting in a sample size of 627 villages.

Since CLTS and SanNut programme implementation would be coordinated at the ward level, we stratified treatment assignment by ward to ensure that each ward had a similar number of treatment and control villages. Within each ward, we randomly assigned half of the villages to receive the SanNut programme (treatment) and half to receive the standard CLTS programme (control). Randomisation was implemented in Stata/IC V.14.0 and documented in .do files. We imported village lists, set the random number seed for reproducibility, generated a random number variable using the runiform() function, sorted the list by ward and the random number variable and assigned the first half of villages within wards to control and the second half to treatment. As such, CHVs, who worked exclusively in their assigned villages, were also effectively randomised to the treatment or control group. If a ward had an odd number of villages, then we assigned the remaining village to the treatment group. Since village lists within wards were randomly sorted, the ‘left-over village’ in wards with an odd number of villages was effectively randomly selected from the pool of evaluation villages so that, within wards, treatment status was orthogonal to village characteristics. We include ward fixed-effects in all analytical models to control for the slightly different probabilities of treatment in odd-number and even-number wards. During data collection, we found that 15 control villages and eight treatment villages did not exist due to errors in the administrative records, resulting in a final sample of 604 villages (309 treatment, 295 control). Villages and households remained balanced on pretreatment covariates: the p value from a joint test of orthogonality on the covariates listed in table 2 is 0.72.

**Table 2:** Balance check, comparison of means across treatment and control villages for household-level variables from the endline survey

	Households in treatment villages	Households in control villages
Likelihood of being below poverty line	43.19	41.57
Caregiver passed Standard 8	0.47	0.51
Age of caregiver	33.07	32.82
Time to fetch water (minutes)	105.31	97.96
Distance to health facility (minutes)	68.40	69.37
Health facility was staffed during last visit	0.81	0.82
Caregiver is in community health group	0.03	0.02
Number of children caregiver has cared for	4.81	4.79
Household has child 0–6 months	0.09	0.10
Household has child 6 months to 2 years	0.44	0.42
Household has child 2–5 years	0.73	0.73

Although we expected the SanNut programme to fail to be implemented in a few treatment villages, in fact the opposite occurred: eight villages, or 3% of all villages assigned to control, were incorrectly treated by SanNut staff. (In one ward, Kitui South, the Public Health Officer who oversaw the implementation of SanNut was different from the one trained by the evaluation team on the distinction between treatment and control villages. As such, incorrect information about the list of villages to be targeted for SanNut was relayed and eight CHVs from control villages were trained and subsequently rolled out the SanNut program.) We use original treatment assignment in all analytical models and report intent-to-treat estimates, though the results do not change substantively if we use treatment-on-the-treated estimator. (See our online supplementary table A1 for treatment-on-the-treated estimates for each of the primary outcomes.)

10.1136/bmjgh-2018-000973.supp1 Supplementary data

**Figure 2:**
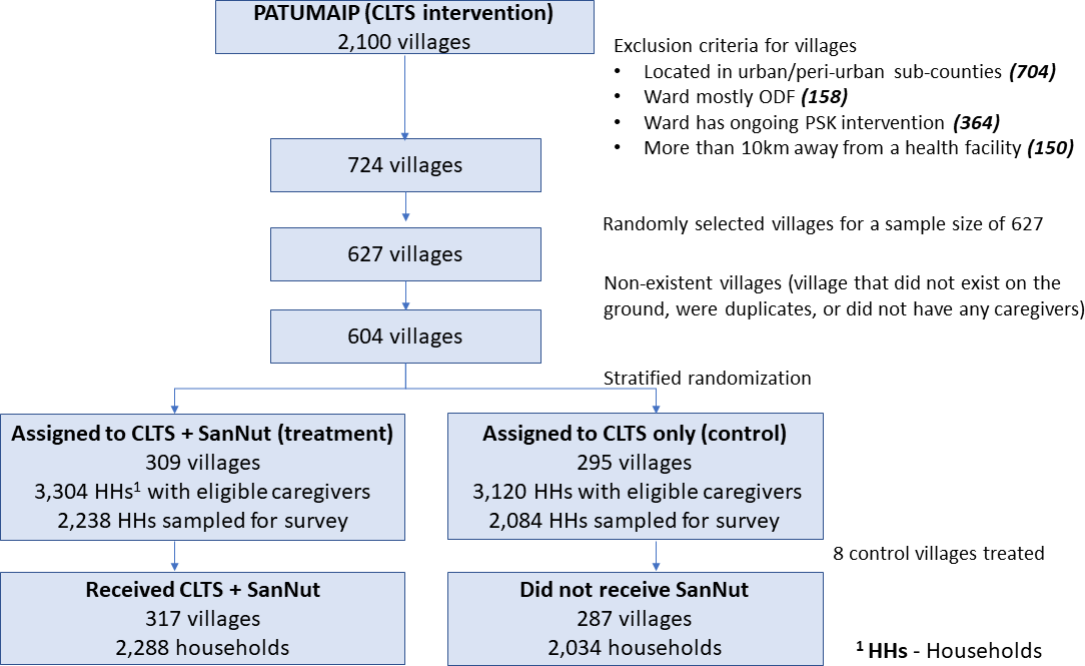
Randomisation and sampling for SanNut study.

To select households within sampled villages we obtained household lists from CHVs. These lists were compiled in all villages in preparation for CLTS and included the number of children below 6 months, the number between 6 months and 2 years and the number between 2 years and 5 years in each household. We obtained these lists in treatment villages immediately prior to SanNut implementation so that CHVs could prepare attendance rolls for caregiver meetings. However, we only obtained household lists in control villages immediately prior to data collection, which was 3 months after obtaining the lists in treatment villages. To ensure that household samples were comparable in treatment and control villages, we ‘trimmed’ the lists to eliminate households that only had a child aged 0–3 months or 57–60 months (and thus would only show up in one group’s list but not the other). The households remaining in the trimmed lists were eligible for inclusion in the study both at the start and end of SanNut implementation (figure 2 shows randomisation and sampling for SanNut study).

The SanNut intervention was targeted at all households with a child under 5 years, but households with children under 2 years were considered high priority by UNICEF since younger children are especially vulnerable to the effects of undernutrition. In order to detect treatment effects within this priority subgroup, we stratified each village list by whether a household had a child under 2 years or a child from 2–5 years (some households had both). To construct our household sample for the endline survey, we randomly selected up to five households in each village from the first sublist of households, ensuring that we had sufficient statistical power to detect effects among households with a child under 2 years. If a village had fewer than five households in this first sublist, all households in that sublist were sampled. After removing households from the second sublist that had already been sampled in the first sublist, we randomly sampled as many households from the second sublist as needed to get eight total households in the village, or all remaining households if the total was fewer than 8. In the final survey sample, the median village contained 7.1 sampled households: 2.1 households with only a child 0–2, 3.3 with only a child 2–5 and 1.8 households with both a child 0–2 and a child 2–5. 8.9% of eligible sampled households in control villages and 9.1% of eligible sampled households in treatment villages were unavailable for the interview and were replaced with other households randomly selected from the eligible pool when possible (Although this left us with only 3.9 households with children 0–2 instead of 5, we still had sufficient statistical power to detect effect sizes of 0.15 SD given the buffer and low rate of non-compliance).

Prior to analysis, we calculated the probability that each eligible household would be selected in the final sample. In order to recover estimates of population average treatment effects for caregivers with children under 5, we weight each observation according to the inverse of the probability of being sampled in all regressions.

### Data collection

We designed a household questionnaire to measure caregiver knowledge, household sanitary and hygiene practices, sanitation outcomes and nutrition outcomes 3 months after the final SanNut activities. Since SanNut was layered onto existing CLTS programming, we also collected data on standard CLTS indicators to assess whether the additional SanNut activities enhanced or detracted from non-child CLTS objectives. The questionnaire consisted of several modules, including a quiz of caregiver knowledge, a survey of caregiver hygiene and sanitation practices, a survey of diarrhoeal incidence of children in the household, enumerator observation of the household environment and enumerator review of children’s health booklets. In summary, we collected data on 15 sanitation and nutrition outcomes, listed in table 3. These 15 outcomes were selected after extensive consultation with UNICEF to determine what evidence was needed to inform their recommendations about scaling the SanNut program.

**Table 3:** Summary of results for all primary outcomes

ID	Outcome	N	Coefficient on treatment	Std error	Control mean
1	Sanitation knowledge index (# questions correct, out of 19 questions). All caregivers.	4322	0.206*	0.110	6.818
2	Safe disposal of child faeces (1=caregiver reports safely disposing of child faeces, 0=otherwise). Restricted to caregivers with a child between 6 months and 2 year.	1951	0.049*	0.022	0.630
3	Caregiver handwashing index (# critical times caregiver reports washing hands, out of 12 possible times). All caregivers.	4322	0.159**	0.061	3.111
4	Functioning latrine (1=functioning latrine observed by enumerator and 0=otherwise). All households.	4322	−0.001	0.012	0.430
5	Latrine use (1=caregiver reports using latrine during last defecation and 0=otherwise). All caregivers.	4322	−0.003	0.014	0.804
6	Courtyard cleanliness index (# courtyard sanitary conditions observed by enumerator, out of six possible checks). All households.	4322	−0.003	0.043	3.565
7	Handwashing station (1=station observed by enumerator and 0=otherwise). All households.	4322	0.057***	0.016	0.148
8	Stocked handwashing station (1=station observed by enumerator stocked with water and soap/ash and 0=otherwise). All households.	4322	0.019**	0.007	0.024
9	Child diarrhoea-self report (1=caregiver reports diarrhoea in last 2 weeks and 0=otherwise). Restricted to children 6 months to 5 years. Excludes 38 eligible children whose caregivers responded don’t know when asked about self-reported diarrhoea incidence.	5481	−0.029*	0.012	0.176
10	Child diarrhoea-stool chart (1=caregiver identifies diarrheal stool type from Bristol Stool Chart and 0=otherwise). Restricted to children 6 months to 5 years. Excludes 753 eligible children whose caregivers responded don’t know when asked to indicate on the stool chart.	4766	−0.014	0.011	0.129
11	Nutrition knowledge index (# questions correct, out of 6 questions). All caregivers	4322	0.114**	0.046	3.535
12	Proper breastfeeding practice (1=caregiver reports exclusive or complementary breastfeeding, depending on child age and 0=otherwise). Restricted to caregivers with a child between 0 and 2 year.	2420	−0.010	0.018	0.772
13	Health facility visit (1=caregiver reports visit to health facility if child was sick and 0=otherwise). Restricted to households with a sick child in the past 2 months	2374	−0.008	0.017	0.833
14	Vitamin A supplementation (1=child health card shows health facility visit for vitamin A supplementation and 0=otherwise). Restricted to children 6 months to 2 year	2115	−0.015	0.015	0.126
15	Deworming (1=child health card shows health facility visit for deworming and 0=otherwise). Restricted to children 1–2 year	1404	0.004	0.012	0.042

All regressions include the control variables listed in table 2, strata fixed effects, sampling weights equal to the inverse probability of selection and standard errors clustered at the village level.

*q<0.10, ** q<0.05, *** q<0.01.

We recruited, trained and managed local enumeration teams. Our teams conducted surveying from April to July 2017, 3 months after the conclusion of SanNut activities. We did not collect baseline data due to time and budgetary constraints. However, the evaluation was powered to detect sufficiently small effects given our sample size under the assumption of no baseline data. Enumerators were not aware of the treatment status of villages that they visited. When an enumeration team reached a village, they would locate sampled households by asking for the head of the household. Once the correct household was identified, enumerators identified the primary caregiver per the household list; in cases where the individual listed was not the primary caregiver, they substituted for the correct caregiver within the same household (this occurred in only 5% of the households surveyed). All survey modules were completed on tablets using SurveyCTO software. We obtained written or verbal informed consent from all study participants.

We registered the SanNut evaluation and preanalysis plan on the American Economic Association’s RCT registry prior to data collection (study ID: AEARCTR-0002019 (https://www.socialscienceregistrysocialscienceregistry.org/trials/2019/history/19731), submitted on 21 February 2017). We also obtained ethical clearance from the Kenya Medical Research Institute (KEMRI) (study ID: Non-KEMRI #547, approved on 31 October 2016) and a research permit from the National Commission for Science, Technology and Innovation (NACOSTI) (study ID: NACOSTI/P/16/57638/12659, approved on 27 July 2016) to conduct the study.

### Analytical model

For each outcome, we estimate the following weighted least squares regression model:

$$Y_{ij}^{}=\beta_{0}^{*}+\beta_{1}^{*}T_{j}+\alpha_{w}^{\prime }\beta^{*}+X_{ij}^{\prime }\beta^{*}+\varepsilon_{ij}^{*}$$

where

- $Y_{\mathrm{ij}}^{}$denotes the outcome variable for household *i* in village *j* (or the caregiver or child in household *i*).
- $T_{j}$denotes the treatment status of village *j* (1=CLTS + SanNut group, 0=CLTS only group).
- $\alpha_{w}^{`}$denotes a vector of dummy variables corresponding to wards (with one ward omitted), which is one when household is in ward *w*, and 0 otherwise.
- $X_{\mathrm{ij}}^{`}$denotes the vector of covariates listed in table 2. The control variables follow from the list specified in our preanalysis plan. We made two slight amendments to this prespecified list due to data limitations: we replaced ‘highest level of education obtained by primary caregiver’ with the binary variable ‘whether caregiver completed Standard 8 or higher’ and we replaced ‘whether health facility was open, staffed and had medications available the last time respondent visited’ with the easier-to-measure ‘whether respondent reported receiving the care that they sought last time they visited the health facility’. Results are similar when these variables are omitted (see online supplementary table A1 for specifications without controls). For households that were missing demographic data, the relevant covariates were set to 0 and dummies for missing covariate data were included in the regression.
- $\varepsilon_{\mathrm{ij}}^{}$denotes the individual error term i, clustered at the village level, which was the level of treatment assignment.
* denotes the sampling weights applied to each household observation, which is equal to the inverse probability of being sampled from all eligible households in the village. Results are similar when households are assigned equal weights (see online supplementary table A1 for ordinary least squares estimates).

Per our preanalysis plan, we analyse the impact of the SanNut programme on the 15 primary outcomes listed in table 3. (All regressions are conducted on the full eligible sample. See online supplementary table A2 for results specific to households with a child 0–2 years, a priority subgroup for UNICEF programming prespecified in our analysis plan.) To account for the multiplicity of hypotheses being tested and to reduce the likelihood of incorrectly rejecting null hypotheses, we control for the false discovery rate (FDR) according to the two-stage linear step-up procedure described in Benjamini *et al*.^18^ This procedure limits the rate of falsely rejecting null hypotheses to a desired level *q*. Rather than set an arbitrary level of *q* for all hypotheses, we follow the algorithm described in Anderson (2008) and perform the procedure for all possible levels of *q* (from 0 to 1 in increments of 0.0001) and record the smallest level *q* when each hypothesis is no longer rejected.^19^ Each estimate’s ‘sharpened q value’ can therefore be interpreted as the expected false discovery rate in the family of outcomes if we reject the null at that level. For convention, in the text, we also report unadjusted p values, but our main effects table and interpretations refer to the FDR-controlled q values. We perform this procedure across all 15 primary outcomes and separately within each of the four indices (sanitation knowledge, caregiver handwashing practices, nutrition knowledge and courtyard cleanliness), which we treat as exploratory analysis to investigate effects on individual components.

## Results

SanNut led to modest improvements in sanitary knowledge and self-reported practices, especially safe handling of child faeces and handwashing. Households in the treatment group were more likely to have a handwashing station that was stocked with soap and water than households in the control group. Caregivers in the treatment group reported lower incidences of child diarrhoea than caregivers in the control group. While SanNut does not appear to have influenced nutritional practices, caregivers in the treatment group were more likely to know that breastfeeding should start immediately after birth. CLTS outcomes, including latrine use and maintenance and general sanitation conditions around the household, were similar in treatment and control groups, suggesting that the impact of SanNut activities neither enhanced nor crowded out CLTS objectives.

### Balance checks

Households sampled for the evaluation were similar across treatment and control villages in terms of demographics and access to health facilities. Table 2 lists household-level variables from the endline survey that are likely to be correlated with health outcomes but should be unrelated to treatment if random assignment was successful. (While some outcomes in table 2 like poverty status or child mortality could plausibly be affected by SanNut in the long-run, we find it unlikely that they would change over the course of a few months and therefore we use these variables as controls in our regression models). These variables are included as controls in all regression models, as prespecified. (Results are similar with and without controls — see Table A1 in the online supplementary appendix 1). Households in treatment and control villages were equally likely to have children 0–6 months, 6 months to 2 years and 2 years to 5 years, indicating that the trimmed administrative lists that were used as sampling frames were comparable across treatment and control villages.

### Household participation in standard CLTS and SanNut activities

The CLTS programme within PATUMAIP was rolled out from June 2016 to January 2017 and was implemented in treatment and control villages at the same time. According to CHV attendance records, caregivers in treatment villages were slightly more likely to attend the CLTS trigger meeting than caregivers in control villages—68.9% of households with children under 5 years attended in treatment villages compared with 63.7% in control villages—though the difference is not statistically significant.

Following the trigger meeting (and after caregiver meetings in SanNut villages), CHVs in both treatment and control villages were expected to visit households with children under 5 years to reinforce the messaging. We asked all sampled households whether they recalled a CHV visit in the past 6 months. Fifty-four per cent of caregivers in treatment villages and 45% of caregivers in control villages recalled such a visit. This difference could indicate an actual increase in CHV visits induced by SanNut, or it could reflect differences in recall if the visits were made more salient by the additional SanNut messaging.

In treatment villages, household participation in the SanNut caregiver meetings was similar to participation in the CLTS triggering meeting, which was comparable to CLTS programmes implemented in other contexts.^8^ According to attendance records collected at SanNut meetings, 70% of eligible caregivers attended the first SanNut meeting (focused on child sanitary practices), 60% attend the second SanNut meeting (focused on child nutrition practices) and 49% attended both. Eighty-one per cent of eligible caregivers attended at least one meeting and 91% of eligible caregivers attended at least one meeting or reported a household visit by a CHV. Twenty-seven per cent of caregivers attended both meetings and reported a household visit by a CHV.

Although health practices and outcomes are better for households with higher levels of SanNut participation, we cannot disentangle the effects of each SanNut activity from endogenous factors that increase SanNut participation. Moreover, households may have been affected by treatment even if they did not report participation in a SanNut activity. This could occur from unreported participation, such as unrecorded attendance in a caregiver meeting or failing to recall a CHV visit. Or households may be affected through ‘spillovers’ from participating neighbours in the form of information sharing, observation of behaviour, or changes in the disease environment.

There is also a possibility of spillovers across villages (in addition to spillovers between neighbors in the same village) due to the proximity of the villages as well as shared community resources such as markets or waterpoints. However, we consider the likelihood of cross-village spillovers to be relatively low given the geographic separation of villages (the average distance between a control household to the nearest treatment village was 2.4 km) and the fact that implementation was conducted at the village level (and CHVs did not overlap). Given these considerations, the estimates reported below are average differences between eligible households in treatment villages and eligible households in control villages irrespective of participation (‘intent-to-treat’ effects).

### Effect of SanNut on primary sanitation and nutrition outcomes

Table 3 contains a summary of the results for all 15 primary outcomes. The statistical tests and results in this table report sharpened q-values, which correct for joint hypothesis tests and dependent outcomes, instead of unadjusted p values.

#### Knowledge about handling of child faeces

Caregivers were asked 19 questions about how to maintain a sanitary environment for their children, the causes of diarrhoeal disease and practices to reduce diarrhoea, and enumerators were instructed to code respondent’s answers from a list of possible options without prompting. The average caregiver in control villages answered 6.8 (36%) of these questions correctly. While the average caregiver in treatment villages only answered 0.2 more questions correctly (q=0.09, p=0.06), she was specifically more knowledgeable about safe handling of child faeces. Caregivers in treatment villages were 5.2 pp or 31% more likely to mention washing hands after handling child faeces as critical (q<0.01, p<0.01). Moreover, caregivers in treatment villages were 3.3 pp or 32% more likely to mention lack of handwashing after handling child faeces as a potential cause of diarrhoea than caregivers in control villages (q=0.09, p=0.03) (see online supplementary table A4 for treatment estimates on each of the 19 components in the sanitation knowledge index).

#### Safe handling of child faeces

Caregivers with a child between 6 months and 2 years were asked how they dispose of their child’s faeces. The disposal method was coded ‘safe’ if it involved discarding faeces in a latrine or burying the faeces in a hole, and ‘unsafe’ if faeces was left in the open, thrown on farmed land or disposed in other ways. Caregivers in treatment villages were 4.9 pp or 7.8% more likely to report safe disposal of child faeces than caregivers in control villages (q=0.05, p=0.02).

Caregivers were asked to list when they typically wash their hands. Once again, enumerators were instructed to code respondent’s answers from a list of possible options without prompting. On average, caregivers in control villages reported washing their hands during 3.1 of the 12 critical activities listed in the survey options, notably after defecating and before eating. While caregivers in treatment villages on average only reported slightly more critical times for washing hands (3.3 times, q=0.05, p=0.01), they were substantially more likely to report washing their hands at times emphasised in SanNut messaging, including after cleaning a child who has defaecated (+5.2 pp or +32%, q<0.01, p<0.01), after handling child faeces (+2.8 pp or +31%, q=0.02, p<0.01) and before feeding their child (+3.1 pp or +21%, q=0.09, p=0.04) (see online supplementary table A5 for treatment estimates on each of the 12 components in the caregiver handwashing index).

#### Handwashing stations and other household sanitary infrastructure

Enumerators conducted an inspection of sanitary conditions and infrastructure in each household, using checklists to record the conditions of latrines, courtyards and handwashing stations. Latrine construction and maintenance are primary objectives of CLTS, and every household must have access to a latrine for the community to achieve ODF status. Eighty per cent of households in the control group had a latrine and reported using it during the last defecation, though only 43% had a functioning latrine. (A functioning latrine was defined as having the following features: the presence of a roof and walls for privacy, the presence slab that is easy to clean, a latrine pit, an aperture cover, and the absence of faeces or flies on the slab). We find no evidence that households in SanNut villages were more or less likely to own a functioning latrine (β=0.01, q=0.546, p=0.442) or to have used a latrine (β=−0.00, q=0.625, p=0.824) than households in control villages. We also find no difference in the courtyard cleanliness index between households in treatment and control villages (β=−0.00, q=0.625, p=0.802), which measured the presence of faeces (adult/child, poultry or animal), trash with flies, animals or stagnant water in courtyards (see online supplementary table A6 for treatment estimates on each of the six components in an index of courtyard cleanliness).

While the importance of handwashing is a standard topic in CLTS messaging, it was emphasised in SanNut caregiver meetings as a critical practice for preventing child diarrhoeal disease. Following SanNut implementation, households in treatment villages were 5.7 pp or 39% more likely to have a place for handwashing (q<0.01, p<0.01), and 1.9 pp or 79% more likely to have a handwashing station stocked with water and soap or ash (q=0.05, p<0.01), than households in control villages.

#### Child diarrhoea

Enumerators asked caregivers if each child in the household aged 6 months to 5 years had suffered from diarrhoea in the past 2 weeks. (Children under 6 months were excluded from this question since they are not expected to eat solid food.) Caregivers in treatment villages reported 2.9 pp or 16% lower incidence of child diarrhoea than caregivers in control villages (q=0.05, p=0.02). Enumerators also showed caregivers a Bristol Stool Chart and asked them to identify each child’s stool type in the past 2 weeks. Respondents were not told which stool types corresponded to diarrhoea. Caregivers in treatment villages were 1.4 pp or 11% less likely to identify that their child’s stool was diarrhoeal on the Bristol Stool Chart, though the difference was not statistically significant (q=0.240, p=0.19).

It is not clear why the apparent effect of SanNut activities on the prevalence of child diarrhoea varies by metric. One possibility is that some caregivers were unsure how to interpret the Bristol Stool Chart and either declined to respond (as did 14% of caregivers), leading to less precise estimates, or made an arbitrary guess, leading to measurement bias (since there were more options to select non-diarrhoeal stool types than diarrhoeal stool types). Notably, the self-reported estimate of diarrheal prevalence in the control group (17.6%) is closer than the Bristol Stool Chart estimate (12.9%) to the prevalence rate in Kitui County according to the latest Kenya Demographic and Health Survey (KDHS 2014) (18%).^15^ However, we cannot reject the null hypothesis that the two-point estimates are the same, and so the difference could also simply reflect statistical noise.

#### Nutritional outcomes

A second objective of the SanNut programme was to highlight the link between child nutritional practices and health outcomes. Caregivers were asked six questions about proper breastfeeding practices. The average caregiver in control villages answered 3.5 of these questions correctly, while the average caregiver in treatment villages answered 3.7 more questions correctly (q=0.05, p=0.01). Although caregivers in treatment villages were 6.6 pp or 10% more likely to know that breastfeeding should start immediately after birth compared with caregivers in control villages (q<0.01, p<0.01), there is no evidence that SanNut increased knowledge about the benefits of breastfeeding or appropriate ages for exclusive and complementary breastfeeding (see online supplementary table A7 for estimates on each of the six components in the nutritionknowledge index).

There is no evidence that SanNut increased self-reported exclusive breastfeeding for children under 6 months or complementary breastfeeding for children 6 months to 2 years (β=−0.01, q=0.58, p=0.58). Finally, there is no evidence that SanNut increased the likelihood of caregivers bringing children to health facilities for nutritional interventions, such as Vitamin A supplementation (β=−0.01, q=0.38, p=0.31) or for deworming treatment (β=0.00, q=0.63, p=0.76).

## Discussion

### Implications of the results

The SanNut programme presents a potentially low-cost, comparatively light-touch opportunity for integrating child-focused sanitation messages into CLTS, with moderate success. SanNut led to modest improvements in child-specific sanitation knowledge, especially about safe handling of child faeces and handwashing. Knowledge gains translated into better sanitary practices: households in the treatment group were more likely to have a handwashing station that was stocked with soap and water, more likely to dispose of child faeces correctly and more likely to report washing hands after handling child faeces or feeding children. There is suggestive evidence that these behaviours led to lower prevalence of child diarrhoea, as reported by caregivers. This finding stands somewhat in contrast to a systematic review of sanitation interventions, as well as a recent RCT of water, sanitation, handwashing and nutritional interventions in Kenya, which found no impact of these interventions on the prevalence of diarrhoeal disease.^7 20^ Our finding highlights an avenue of further research on the potential for child-focused behaviour change interventions like SanNut to improve child health.

In contrast to our sanitation results, we found limited evidence that SanNut improved knowledge or practices around breastfeeding or health facility visits for nutritional check-ups. This may in part be due to the fact that nutritional knowledge and practices were already at high levels and had relatively little room for improvement. In control villages, 84% of caregivers knew that children should be exclusively breastfed in the first 6 months, and 77% of caregivers reported following correct breastfeeding guidelines (exclusive breastfeeding 0–6 months and complementary breastfeeding 6–24 months). At these levels, it may be particularly difficult to change caregiver practices on the margin.

The nutrition component of SanNut also faced some implementation challenges that may have further reduced its potential for impact. Attendance at the nutrition-focused caregiver meeting was 13% lower than attendance at the sanitation-focused caregiver meeting. Among caregivers in SanNut villages who reported a CHV visit, 20% recall the CHV discussing exclusive breastfeeding, 30% recall the CHV discussing complementary breastfeeding and just 13% recall the CHV discussing the importance of routine visits to the health facility. Addressing these implementation challenges in future iterations of the SanNut programme could lead to improvements in nutritional practices.

Overall, our results suggest that child-focused messaging can potentially be integrated into existing CLTS programming, though this integration was more successful for topics that were closer to CLTS objectives (limiting faecal contamination, handwashing) than for topics that were more disparate (breastfeeding, vitamin A supplementation, deworming).

### Limitations and areas for further research

We acknowledge several limitations to our study and highlight areas for further research.

First, our study was designed to measure the effect of assigning a package of SanNut activities, including a sanitation-focused caregiver meeting, a nutrition-focused caregiver meeting and additional messaging in CHV visits, to villages receiving CLTS programming. We are not able to disentangle the individual effectiveness of each of these components or interaction effects between them. Future evaluations could highlight the relative importance of each activity in the programme’s impact. In particular, further research is needed to assess the impact of integrated sanitation and nutrition programme that emphasise the nutrition component more than SanNut did. Recent trials in Kenya and Bangladesh found that nutrition interventions improved growth of children, but that there were no additional benefits from the integration of water, sanitation and handwashing and nutrition interventions.^20 21^ Since SanNut’s nutrition messaging was relatively limited, further research should explore whether a wider suite of high-impact nutrition interventions would be more appropriate in areas with high stunting rates such as Kitui and what levels of integration are appropriate for such contexts.

Second, our estimates could be biassed if treatment households failed to be treated or if control households were treated directly (non-compliance) or benefitted indirectly from nearby treated households (spillovers). We closely monitored implementation and found that eight of the 295 control villages incorrectly received the SanNut intervention. In the online supplementary table A1, we instrument actual treatment with assigned treatment and estimate treatment-on-the-treated effects, which do not substantively change any of our results. Beyond these cases of non-compliance, we believe that spillovers were largely mitigated since treatment assignment occurred at the village level, CHVs were assigned exclusively to one village and villages are geographically separate, though we cannot rule out this possibility of unobserved spillovers. If any control households benefitted from the SanNut intervention, then our estimates would likely be biassed towards zero.

Third, our study relied on several outcomes reported by caregivers, including handwashing practices, disposal of child faeces, breastfeeding practice and the occurrence of child diarrhoea. Since households were aware of the SanNut intervention happening (or not happening) in their village, it is possible that these outcomes were subject to self-reporting bias and that actual impacts on health behaviour were smaller than our estimates. Further research is needed to determine whether these proximal outcomes translate into actual behaviour change.

Fourth, our study measured the effect of SanNut 3 months after the programme was implemented. Further research is needed to explore the long-term impact of integrated interventions on child stunting and under-five mortality, particularly for information-based interventions like SanNut. On the one hand, the impact of the programme on behaviour change may fall over time as the information becomes less salient; on the other hand, the impact may increase as information is shared and behaviours are adopted within peer networks.^22^

Finally, our study measured the effects of SanNut in one region with especially critical rates of child undernutrition. Forty-six per cent of children in Kitui County are stunted, compared with 26% of children nationwide. Since SanNut was only piloted in a single context, further research is needed to assess whether its benefits replicate in other settings, such as areas with lower (and higher) rates of undernutrition, different types of malnutrition or different models of CLTS implementation. In particular, further research is needed to determine whether SanNut is an effective supplement to CLTS programming in contexts with lower levels of child undernutrition or with different types of undernutrition such as wasting, especially in drought-affected areas.

## References

[1] Black RE, Victora CG, Walker SP, et al Maternal and child undernutrition and overweight in low-income and middle-income countries. Lancet 2013;382:427–51. 10.1016/S0140-6736(13)60937-X 23746772

[2] Guerrant RL, DeBoer MD, Moore SR, et al The impoverished gut--a triple burden of diarrhoea, stunting and chronic disease. Nat Rev Gastroenterol Hepatol 2013;10:220–9. 10.1038/nrgastro.2012.239 23229327 PMC3617052

[3] Korpe PS, Petri WA, William AP Environmental enteropathy: critical implications of a poorly understood condition. Trends Mol Med 2012;18:328–36. 10.1016/j.molmed.2012.04.007 22633998 PMC3372657

[4] Lin A, Arnold BF, Afreen S, et al Household environmental conditions are associated with enteropathy and impaired growth in rural Bangladesh. Am J Trop Med Hyg 2013;89:130–7. 10.4269/ajtmh.12-0629 23629931 PMC3748469

[5] Garn JV, Sclar GD, Freeman MC, et al The impact of sanitation interventions on latrine coverage and latrine use: a systematic review and meta-analysis. Int J Hyg Environ Health 2017;220 329–40. 10.1016/j.ijheh.2016.10.001 27825597 PMC5414716

[6] Sclar GD, Penakalapati G, Amato HK, et al Assessing the impact of sanitation on indicators of fecal exposure along principal transmission pathways: a systematic review. Int J Hyg Environ Health 2016;219:709–23. 10.1016/j.ijheh.2016.09.021 27720133

[7] Freeman MC, Garn JV, Sclar GD, et al The impact of sanitation on infectious disease and nutritional status: a systematic review and meta-analysis. Int J Hyg Environ Health 2017;220:928–49. 10.1016/j.ijheh.2017.05.007 28602619

[8] Alzua ML, Pickering AJ, Djebbari H Final Report: Impact Evaluation of Community-led Total Sanitation (CLTS) in Rural Mali. UNICEF, 2015.

[9] Patil SR, Arnold BF, Salvatore AL, et al The effect of India's total sanitation campaign on defecation behaviors and child health in rural Madhya Pradesh: a cluster randomized controlled trial. PLoS Med 2014;11:e1001709 10.1371/journal.pmed.1001709 25157929 PMC4144850

[10] Bhandari N, Bahl R, Mazumdar S, et al Effect of community-based promotion of exclusive breastfeeding on diarrhoeal illness and growth: a cluster randomised controlled trial. Lancet 2003;361:1418–23. 10.1016/S0140-6736(03)13134-0 12727395

[11] Haider R, Ashworth A, Kabir I, et al Effect of community-based peer counsellors on exclusive breastfeeding practices in Dhaka, Bangladesh: a randomised controlled trial [see commments]. Lancet 2000;356:1643–7. 10.1016/S0140-6736(00)03159-7 11089824

[12] Morrow AL, Guerrero ML, Shults J, et al Efficacy of home-based peer counselling to promote exclusive breastfeeding: a randomised controlled trial. Lancet 1999;353:1226–31. 10.1016/S0140-6736(98)08037-4 10217083

[13] Aidam BA, Pérez-Escamilla R, Lartey A Lactation counseling increases exclusive breast-feeding rates in Ghana. J Nutr 2005;135:1691–5. 10.1093/jn/135.7.1691 15987851

[14] Bhutta ZA, Das JK, Rizvi A, et al Evidence-based interventions for improvement of maternal and child nutrition: what can be done and at what cost? Lancet 2013;382:452–77. 10.1016/S0140-6736(13)60996-4 23746776

[15] Kenya National Bureau of Statistics, Ministry of Health/Kenya, National AIDS Control Council/Kenya Kenya demographic and health survey 2014. Rockville, MD, USA: Kenya National Bureau of Statistics, 2015.

[16] Abramovsky L, Augsburg B, Oteiza F Sustainable total sanitation – Nigeria. The Institute for Fiscal Studies, 2015.

[17] Lewycka S, Mwansambo C, Kazembe P, et al A cluster randomised controlled trial of the community effectiveness of two interventions in rural Malawi to improve health care and to reduce maternal, newborn and infant mortality. Trials 2010;11:88 10.1186/1745-6215-11-88 20849613 PMC2949851

[18] Benjamini Y, Krieger AM, Yekutieli D Adaptive linear step-up procedures that control the false discovery rate. Biometrika 2006;93:491–507. 10.1093/biomet/93.3.491

[19] Anderson ML Multiple inference and gender differences in the effects of early intervention: a reevaluation of the abecedarian, perry preschool, and early training projects. J Am Stat Assoc 2008;103:1481–95. 10.1198/016214508000000841

[20] Null C, Stewart CP, Pickering AJ, et al Effects of water quality, sanitation, handwashing, and nutritional interventions on diarrhoea and child growth in rural Kenya: a cluster-randomised controlled trial. Lancet Glob Health 2018;6:e316–e329. 10.1016/S2214-109X(18)30005-6 29396219 PMC5809717

[21] Luby SP, Rahman M, Arnold BF, et al Effects of water quality, sanitation, handwashing, and nutritional interventions on diarrhoea and child growth in rural Bangladesh: a cluster randomised controlled trial. Lancet Glob Health 2018;6:e302–e315. 10.1016/S2214-109X(17)30490-4 29396217 PMC5809718

[22] Duflo E, Saez E The role of information and social interactions in retirement plan decisions: evidence from a randomized experiment. Q J Econ 2003;118:815–42. 10.1162/00335530360698432

