# Supplementary material for: Can child-focused sanitation and nutrition programming improve health practices and outcomes? Evidence from a randomised controlled trial in Kitui County, Kenya
Source: BMJ Glob Health. 2019 Jan 5;4(1):e000973. doi: 10.1136/bmjgh-2018-000973 (PMC6326299; doi:10.1136/bmjgh-2018-000973)
Supplement: Supplementary data [file bmjgh-2018-000973supp001.pdf]

Appendix to "Can child-focused sanitation and nutrition programming improve health practices and outcomes? Evidence from a Randomized Controlled Trial in Kitui County, Kenya", 2018

Table A1: Primary outcomes, treatment effect coefficients from alternative specifications

ID	*Outcome*	*1: Main spec*	*2: No controls*	*3: Equal weights*	*4: No controls, Equal weights*	*5. Treatment-on-the-treated*
1	Sanitation knowledge index (# questions correct, out of 19 questions). All caregivers.	0.206*	0.195	0.190	0.185	0.211*
2	Safe disposal of child faeces (1 = caregiver reports safely disposing of child faeces, 0 otherwise). Restricted to caregivers with a child between 6mo and 2yr.	0.049*	0.049*	0.047*	0.048*	0.051**
3	Caregiver handwashing index (# critical times caregiver reports washing hands, out of 12 possible times). All caregivers.	0.159**	0.159*	0.143*	0.147*	0.163**
4	Functioning latrine (1 = functioning latrine observed by enumerator, 0 otherwise). All households.	-0.001	-0.002	-0.006	-0.005	-0.001
5	Latrine use (1 = caregiver reports using latrine during last defecation, 0 otherwise). All caregivers.	-0.003	-0.004	-0.007	-0.007	-0.003
6	Courtyard cleanliness index (# courtyard sanitary conditions observed by enumerator, out of 6 possible checks). All households.	-0.003	-0.003	0.004	0.009	-0.003
7	Handwashing station (1 = station observed by	0.057***	0.056***	0.054***	0.055***	0.058***

	enumerator, 0 otherwise). All households.					
8	Stocked handwashing station (1 = station observed by enumerator stocked with water and soap/ash, 0 otherwise). All households.	0.019**	0.019*	0.020**	0.020**	0.019**
9	Child diarrhoea-self report (1 = caregiver reports diarrhoea in last two weeks, 0 otherwise). Restricted to children 6 months to 5 years. Excludes 38 eligible children whose caregivers responded Don't Know when asked about self-reported diarrhoea incidence.	-0.029*	-0.028*	-0.035***	-0.035***	-0.029**
10	Child diarrhoea-stool chart (1 = caregiver identifies diarrheal stool type from Bristol Stool Chart, 0 otherwise). Restricted to children 6 months to 5 years. Excludes 753 eligible children whose caregivers responded Don't Know when asked to indicate on the stool chart.	-0.014	-0.014	-0.018	-0.019	-0.014
11	Nutrition knowledge index (# questions correct, out of 6 questions). All caregivers	0.114**	0.110*	0.106**	0.105*	0.116**
12	Proper breastfeeding practice (1 = caregiver reports exclusive or complementary breastfeeding, depending on child age, 0 otherwise). Restricted to caregivers with a child between 0 and 2yr.	-0.010	-0.008	-0.002	0.001	-0.010
13	Health facility visit (1 = caregiver reports visit to health facility if child was sick, 0 otherwise). Restricted to households with a sick child in the past two months	-0.008	-0.007	-0.012	-0.012	-0.009

14	Vitamin A supplementation (1 = child health card shows health facility visit for Vitamin A supplementation, 0 otherwise). Restricted to children 6mo to 2yr	-0.015	-0.015	-0.007	-0.007	-0.015
15	Deworming (1 = child health card shows health facility visit for deworming, 0 otherwise). Restricted to children 1 to 2yr	0.004	0.004	-0.003	-0.003	0.004

"1. Main spec": Controls for the covariates listed in Table 2 in the main report and weights observations by the inverse probability of being sampled. "2. No controls": Omits covariates except for strata (ward-level) dummies. "3. Equal weights": Removes probability weights, effectively weighting each observation equally. "4. No controls, Equal weights": Omits covariates and weights each observation equally. "5. Treatment-on-the-treated": Instruments actual treatment with assigned treatment to produce local average treatment effect estimates, adjusting for the 8 control villages that did not comply with their treatment assignment.

** q<0.10, ** q<0.05, *** q<0.01*

Table A2: Primary outcomes for subgroup of households with a child 0 to 2 years[1]

ID	*Outcome*	*N*	*Coef on Treatment*	*Std Error*	*Control mean*
1	Sanitation knowledge index (# questions correct, out of 19 questions). All caregivers.	2,327	0.228	0.132	6.761
2	Safe disposal of child faeces (1 = caregiver reports safely disposing of child faeces, 0 otherwise). Restricted to caregivers with a child between 6mo and 2yr.	1,951	0.049	0.022	0.630
3	Caregiver handwashing index (# critical times caregiver reports washing hands, out of 12 possible times). All caregivers.	2,327	0.165	0.078	3.177
4	Functioning latrine (1 = functioning latrine observed by enumerator, 0 otherwise). All households.	2,327	0.004	0.015	0.421
5	Latrine use (1 = caregiver reports using latrine during last defecation, 0 otherwise). All caregivers.	2,327	-0.008	0.018	0.799

[1] Children 0 to 2 years were a priority subgroup for UNICEF's programming, and so our pre-analysis plan called for additional analysis of outcomes for households with children in this age group.

6	Courtyard cleanliness index (# courtyard sanitary conditions observed by enumerator, out of 6 possible checks). All households.	2,327	0.011	0.052	3.567
7	Handwashing station (1 = station observed by enumerator, 0 otherwise). All households.	2,327	0.046	0.018	0.147
8	Stocked handwashing station (1 = station observed by enumerator stocked with water and soap/ash, 0 otherwise). All households.	2,327	0.015	0.008	0.021
9	Child diarrhoea-self report (1 = caregiver reports diarrhoea in last two weeks, 0 otherwise). Restricted to children 6 months to 2 years. Excludes 38 eligible children whose caregivers responded Don't Know when asked about self-reported diarrhoea incidence.	3,194	-0.031	0.018	0.226
10	Child diarrhoea-stool chart (1 = caregiver identifies diarrheal stool type from Bristol Stool Chart, 0 otherwise). Restricted to children 6 months to 2 years. Excludes 753 eligible children whose caregivers responded Don't Know when asked to indicate on the stool chart.	2,853	-0.023	0.014	0.144
11	Nutrition knowledge index (# questions correct, out of 6 questions). All caregivers	2,327	0.208***	0.057	3.476
12	Proper breastfeeding practice (1 = caregiver reports exclusive or complementary breastfeeding, depending on child age, 0 otherwise). Restricted to caregivers with a child between 0 and 2yr.	2,420	-0.010	0.018	0.772
13	Health facility visit (1 = caregiver reports visit to health facility if child was sick, 0 otherwise). Restricted to households with a sick child in the past two months	1,345	-0.016	0.021	0.855
14	Vitamin A supplementation (1 = child health card shows health facility visit for Vitamin A supplementation, 0 otherwise). Restricted to children 6mo to 2yr	2,115	-0.015	0.015	0.126

15	Deworming (1 = child health card shows health facility visit for deworming, 0 otherwise). Restricted to children 1 to 2yr	1,404	0.004	0.012	0.042

All regressions include the control variables listed in Table 2 in the main report, strata fixed effects, sampling weights equal to the inverse probability of selection, and standard errors clustered at the village-level.

** q<0.10, ** q<0.05, *** q<0.01*

Table A3: Treatment effect coefficients for alternative index aggregation methods

ID	*Outcome index*	*1: # correct or observed (main spec)*	*2: % correct or observed*	*3: Standardized components, equal weighting*	*4: Standardized components, inverse covariance weighting*
1	Sanitation knowledge index (out of 19 questions). All caregivers.	0.206*	0.011*	0.037*	0.082*
2	Caregiver handwashing index (out of 12 critical times for handwashing). All caregivers.	0.159**	0.013**	0.046**	0.108**
3	Courtyard cleanliness index (out of 6 possible checks of courtyard sanitary conditions). All households.	-0.003	-0.002	-0.010	-0.002
4	Nutrition knowledge index (out of 6 questions). All caregivers	0.114**	0.019**	0.046**	0.103**

"1. # correct or observed (main spec)": The number of items that the respondents answered correctly (sanitation index, caregiver handwashing index, and nutrition index) or that were observed by the enumerator (courtyard cleanliness index). "2. % correct or observed": Percent of items correct/observed out of all items in the index. "3. Standardized components, equal weighting": Individual index components are normalized, using the control group mean and standard deviation, and then averaged with equal weights to form the index. "4. Standardized components, inverse covariance weighting": Individual index components are normalized, using the control group mean and standard deviation, and then weighted according to the inverse of the covariance matrix of all index components. This method follows Anderson (2008), also cited in the main report, and gives less weight to outcomes that are highly correlated.
All regressions include the control variables listed in Table 2 in the main report, strata fixed effects, sampling weights equal to the inverse probability of selection, and standard errors clustered at the village-level.

** q<0.10, ** q<0.05, *** q<0.01*

Table A4: Treatment effects on individual index components – Sanitation knowledge (N = 4,322)

ID	*Outcome*	*Coef on Treatment*	*Std Error*	*Control mean*

What are ways to maintain a sanitary environment inside the home to prevent contamination of food from fecal matter?				
1	Build/use a latrine	0.039**	0.014	0.130
2	Store/cover food	-0.041**	0.014	0.837
3	Safe disposal of child faeces	0.021	0.013	0.125
4	Safe disposal of animal faeces	0.043**	0.013	0.086
5	Handwashing after defecation	0.016	0.013	0.092
6	Use clean utensils when cooking/eating	0.021	0.010	0.051
7	Handwashing before handling food	0.026	0.015	0.114
What are some practices that could cause a person to get diarrhoea?				
8	No handwashing before eating	-0.013	0.017	0.384
9	No handwashing after defecation	0.006	0.016	0.285
10	No handwashing after cleaning child's stool	0.033*	0.015	0.103
11	Drinking untreated water	0.024	0.018	0.376
12	Improper cooking of food	-0.020	0.017	0.306
13	Not cleaning or washing vegetables	-0.001	0.013	0.161
14	Eating food contaminated by flies	0.010	0.008	0.049
What are some practices that could prevent getting diarrhoea from water?				
15	Treating water by boiling, use chlorine, or solar water disinfecting	0.001	0.012	0.877
When do you think are the critical times to wash your hands?				
16	For preparing food and eating	0.006	0.012	0.860
17	After using latrine	-0.021	0.012	0.864
18	After handling child faeces	0.052***	0.015	0.169
What is the safest way to dispose of your child's stool?				
19	Latrine use, putting faeces into toilet, or burying	0.004	0.007	0.948

All regressions include the control variables listed in Table 2 in the main report, strata fixed effects, sampling weights equal to the inverse probability of selection, and standard errors clustered at the village-level.

** q<0.10, ** q<0.05, *** q<0.01*

Table A5: Treatment effects on individual index components – Caregiver handwashing (N = 4,322)

ID	*Outcome*	*Coef on Treatment*	*Std Error*	*Control mean*
When do you regularly wash your hands?				
1	When hands are dirty	-0.020	0.019	0.379
2	When returning to house from work or outside	0.000	0.013	0.129
3	Before eating	-0.006	0.016	0.719
4	After eating	0.004	0.016	0.282
5	Before preparing food	0.051**	0.017	0.299
6	After defecation	-0.005	0.012	0.855
7	After touching animals	0.010	0.006	0.029
8	After handling animal feces	0.008	0.004	0.013
9	After taking care of sick people	0.003	0.002	0.002
10	After cleaning child who has defecated	0.053***	0.014	0.165
11	After handling child faeces	0.028**	0.010	0.091
12	Before feeding the child	0.033*	0.014	0.150

All regressions include the control variables listed in Table 2 in the main report, strata fixed effects, sampling weights equal to the inverse probability of selection, and standard errors clustered at the village-level.

** q<0.10, ** q<0.05, *** q<0.01*

Table A6: Treatment effects on individual index components – Courtyard cleanliness (N = 4,322)

ID	*Outcome*	*Coef on Treatment*	*Std Error*	*Control mean*
Enumerator: Walk around the courtyard and mark if you observe any of the following				

1	Absence of human faeces	-0.006	0.005	0.983
2	Absence of stagnant water	0.000	0.005	0.972
3	Absence of animal faeces	-0.007	0.017	0.223
4	Absence of trash with flies	-0.020	0.016	0.834
5	Whether courtyard appears swept	0.019	0.020	0.446
6	Absence of animals or poultry	0.005	0.012	0.130

All regressions include the control variables listed in Table 2 in the main report, strata fixed effects, sampling weights equal to the inverse probability of selection, and standard errors clustered at the village-level.

** q<0.10, ** q<0.05, *** q<0.01*

Table A7: Treatment effects on individual index components – Nutrition knowledge (N = 4,322)

ID	***Outcome***	***Coef on Treatment***	***Std Error***	***Control mean***
What are the benefits of feeding your child breastmilk?				
1	Nutritious to baby	-0.016	0.016	0.758
2	Prevents disease/infections	0.023	0.018	0.522
3	Bonding between mother and baby	0.005	0.005	0.023
How long after birth should a child be put to breast?				
4	Immediately after birth	0.064***	0.016	0.694
For how many months should a child be fed on breast milk without being given anything else?				
5	Exclusive breastfeeding for 6 months	0.025	0.017	0.842
At what age in months should one introduce solid food to a baby?				
6	Complementary breastfeeding at 7 months	0.013	0.019	0.697

All regressions include the control variables listed in Table 2 in the main report, strata fixed effects, sampling weights equal to the inverse probability of selection, and standard errors clustered at the village-level.

** q<0.10, ** q<0.05, *** q<0.01*
